# How is adults’ screen time behaviour influencing their views on screen time restrictions for children? A cross-sectional study

**DOI:** 10.1186/s12889-016-2789-3

**Published:** 2016-03-01

**Authors:** Stephanie Schoeppe, Amanda L. Rebar, Camille E. Short, Stephanie Alley, Wendy Van Lippevelde, Corneel Vandelanotte

**Affiliations:** Physical Activity Research Group, Central Queensland University, School of Human Health and Social Sciences, Building 18 Bruce Highway, North Rockhampton, QLD 4702 Australia; Freemasons Foundation Centre for Men’s Health, South Australian Health and Medical Research Institute, The University of Adelaide, North Terrace, Adelaide, Australia; Department of Public Health, Ghent University, De Pintelaan 185 – 4K3 room 036, 9000 Ghent, Belgium

**Keywords:** Adult, Parent, Children, Television, Computer, Screen time, Rules, Restrictions, Sedentary behaviour

## Abstract

**Background:**

High screen time in children and its detrimental health effects is a major public health problem. How much screen time adults think is appropriate for children remains little explored, as well as whether adults’ screen time behaviour would determine their views on screen time restrictions for children. This study aimed to investigate how adults’ screen time behaviour influences their views on screen time restrictions for children, including differences by gender and parental status.

**Methods:**

In 2013, 2034 Australian adults participated in an online survey conducted by the Population Research Laboratory at Central Queensland University, Rockhampton. Adult screen time behaviour was assessed using the Workforce Sitting Questionnaire. Adults reported the maximum time children aged between 5–12 years should be allowed to spend watching TV and using a computer. Ordinal logistic regression was used to compare adult screen time behaviour with views on screen time restrictions for children.

**Results:**

Most adults (68 %) held the view that children should be allowed no more than 2 h of TV viewing and computer use on school days, whilst fewer adults (44 %) thought this screen time limit is needed on weekend days. Women would impose higher screen time restrictions for children than men (*p* < 0.01). Most adults themselves spent > 2 h on watching TV and using the computer at home on work days (66 %) and non-work days (88 %). Adults spending ≤ 2 h/day in leisure-related screen time were less likely to permit children > 2 h/day of screen time. These associations did not differ by adult gender and parental status.

**Conclusions:**

Most adults think it is appropriate to limit children’s screen time to the recommended ≤ 2 h/day but few adults themselves adhere to this screen time limit. Adults with lower screen use may be more inclined to limit children’s screen time. Strategies to reduce screen time in children may also need to target adult screen use.

## Background

Screen time, such as TV viewing and computer use, has been associated with overweight and obesity in children and adolescents [1, 2]. High prevalence of screen time and its detrimental health effects for children is a major public health problem [3]. In Australia, 71 % of children aged 5–17 years exceed recommended limits of screen time (≤2 h/day of television, seated electronic games and computer use) [4]. Limiting TV viewing and computer use in children is important as screen time tends to increase during adolescence [5] and remains highly prevalent in adulthood, exacerbating the problem of sedentariness, physical inactivity and obesity [6].

Adults including parents, grand-parents and other caregivers can influence children’s screen time by restricting TV viewing and computer use [7]. Usually, adults recognise the importance of limiting screen time but fail to impose screen time restrictions for children [8]. Moreover, adults often model excessive TV viewing and computer use themselves [8], which may impede their willingness and effectiveness to enforce screen time restrictions for children. Despite increasing research on adults’ role in limiting children’s screen time, little is known about how much screen time adults think is appropriate for children, and whether this corresponds to established screen time recommendations for children [9]. Further, it is unclear to what extent adults’ own screen time behaviour would determine their views on screen time restrictions for children. Finally, most previous research in this area has concentrated solely on parents [10]; however, many other adults who supervise children, such as grand-parents, aunts and uncles, friends and child care workers, also play an important role in limiting children’s screen time [11]. Hence, it is worth exploring this topic beyond the traditional parent–child relationship.

This study aimed to investigate how adults’ screen time behaviour influences their views on screen time restrictions for children, including differences by gender and parental status. This information may improve the development of potentially more effective family-based interventions to reduce screen time in children.

## Methods

### Study population

Study participants were panel members of the Australian Health and Social Science project, recruited through annual population surveys conducted by the Population Research Laboratory at Central Queensland University. Between October and November 2013, 3901 randomly selected panel members were invited to participate in an online survey. Of these, 2034 respondents (52.1 %) across all States and Territories of Australia completed the survey [12]. Participants provided informed consent and the Human Ethics Committee at Central Queensland University approved the study (H13/09-163).

### Measures

#### Socio-demographic variables

Socio-demographic variables measured included age, sex, parental status (parent of children 0–12 years, parent of children ≥ 13 years, non-parent) and level of education (high school, trade certificate/diploma, university degree).

#### Adult screen time behaviour

Adult screen time behaviour was assessed using two items from the Workforce Sitting Questionnaire developed by Chau et al. [13]. The items measure discretionary screen time during leisure (excluding screen time at work), and have shown acceptable test-retest reliability (ICC = 0.56–0.91) and criterion validity against accelerometry (*r* = 0.23–0.34) [13]. Adults were asked to report their time spent sitting while 1) watching TV and 2) using a computer at home for leisure activities (e.g., email, games, information, chatting) on a work day and a non-work day in the last 7 days. Total screen time on work days and non-work days, respectively, was the sum of time adults spent watching TV and using the computer on a work day/non-work day. The variables ‘total screen time on a work day’ and ‘total screen time on a non-work day’ were dichotomised into ‘≤2 h/day’ and ‘>2 h/day’ to assess whether adults themselves would exceed screen time limits recommended for children [9].

#### Adult views on screen time restrictions for children

Adult views on screen time restrictions for children were assessed using two items. Adults were asked ‘What is the maximum time children aged between 5–12 years should be allowed to spend watching TV?’ and ‘What is the maximum time children aged between 5–12 years should be allowed to spend using a computer at home for fun (e.g. games, emails, chatting, surfing the internet)?’ The questions were asked separately for a usual school day and a usual weekend day. The age group 5–12 years was chosen because children’s screen time habits tend to develop during this age [14]. Response options for both questions were no more than 15 min, no more than 30 min, no more than 1 h, no more than 2 h, no more than 3 h, no more than 4 h and 5 h or more. Based on distributions, responses for maximum screen time were collapsed into the categories no more than 30 min (≤30 min), no more than 1 h (≤1 h), no more than 2 h (≤2 h), and 2 h or more (>2 h). Total screen time adults would allow children on school days and weekend days, respectively, was the sum of maximum time adults would allow children for watching TV and using the computer on a usual school day/weekend day.

### Statistical analyses

Chi-square and independent t-tests were performed to assess differences in socio-demographic variables, screen time behaviour and views on screen time restrictions for children between men and women, and included and excluded participants. All variables were checked for normal distribution by examining descriptive statistics, boxplots and histograms. Ordinal logistic regression was used to assess associations between adults’ screen time behaviour and their views on screen time restrictions for children. Predictor variables were adults’ ‘total screen time on a work day’ and ‘total screen time on a non-work day’. Outcome variables were ‘screen time adults would allow children on school days’ and ‘screen time adults would allow children on weekend days’ (reference category: > 2 h a day). Four ordered logit models were run by combining one of the two predictor variables with one of the two outcome variables. An interaction term was entered in the ordered logit models to test for differences in associations by gender and parental status. Analyses were adjusted for adult age, sex, parental status and level of education (covariates). Variance inflation factors, R2-square values and parameter estimates were inspected to ensure there was no multicollinearity amongst predictor variables and covariates. Associations are presented using proportional odds ratios (ORs), 95 % confidence intervals (CIs) and *p*-values with significance levels set at *p* < 0.05. Participants with missing data across all variables were excluded from analyses (*n* = 498). Analyses were performed in IBM SPSS Statistics (version 22.0).

## Results

Descriptive statistics for the socio-demographic variables, adult screen time behaviour and adult views on screen time restrictions for children are presented in Table 1. Adults’ mean age was 56.0 (SD = 13.7) years, 58 % were male, 82 % were parents and 44 % had a university degree education. There were no significant differences in sex, screen time behaviour or views on screen time restrictions for children between participants included and excluded from the analyses. However, compared to included participants, excluded participants were older (55.4 vs 59.0 years; *p* < 0.001), had lower levels of education (high school: 9 vs 15 %; *p* = 0.001), and were more likely to be parents of older children (child ≥ 13 years: 27 vs 39 %; *p* = 0.003). The majority of adults spent > 2 h watching TV and using the computer at home on work days (66 %) and non-work days (88 %). More men than women engaged in > 2 h of screen time on work days (71 vs 62 %; *p* = 0.022). Most adults (68 %) held the view that children aged 5–12 years should spend no more than 2 h on TV viewing and computer use on school days, whilst fewer adults (44 %) thought this screen time limit is needed on weekend days. Compared to men, women were more inclined to restrict children’s screen time on school days and weekend days (*p* < 0.01). The results of the ordinal regression analyses examining associations between adults’ screen time behaviour and the screen time adults thought is appropriate for children are presented in Table 2. Adults who engaged ≤ 2 h/day in leisure-related screen time were less likely to allow children > 2 h of screen time on school days (OR = 0.45, 95 % CI: 0.20–1.00) and weekend days (OR = 0.33, 95 % CI: 0.14–0.75). There were no significant differences in the observed associations by gender and parental status.

**Table 1:** Descriptive statistics (*N* = 2034)^a^

	All	Male	Female	*p*-value
Age (mean (SD))	56.0 (13.7)	58.2 (13.7)	53.9 (13.3)	0.000
Sex (%)	100.0	47.7	52.3	
Parental status (%)				0.225
Parent (child 0–12 years)	15.7	14.4	17.0	
Parent (child ≥ 13 years)	65.8	67.5	64.1	
Non-parent	18.5	18.1	18.9	
Education (%)				0.019
High school	23.4	21.2	25.4	
Trade certificate/Diploma	32.4	35.3	29.8	
University degree	44.2	43.5	44.8	
Adult screen time behaviour				
Work day				
≤ 2 h (%)	33.8	29.5	37.9	0.022
> 2 h (%)	66.2	70.5	62.1	
Minutes per day (mean (SD))	181.9 (181.7)	188.6 (182.3)	175.1 (179.1)	0.287
Non-work day				
≤ 2 h (%)	11.8	10.4	13.3	0.079
> 2 h (%)	88.2	89.6	86.7	
Minutes per day (mean (SD))	336.45 (233.0)	345.8 (227.8)	329.4 (239.8)	0.166
Adult views on screen time restrictions for children (%)				
School day				0.000
≤ 30 min	2.4	2.2	2.7	
≤ 1 h	16.9	13.9	19.9	
≤ 2 h	48.4	47.0	49.6	
> 2 h	32.2	36.0	27.8	
Weekend day				0.002
≤ 30 min	1.8	2.5	1.1	
≤ 1 h	9.2	8.5	9.4	
≤ 2 h	32.8	29.3	36.2	
> 2 h	56.2	59.7	53.2	

^a^2013 Australian Health and Social Science panel study, Rockhampton

**Table 2:** Associations between adult screen time behaviour and screen time adults would allow children^a^

	Screen time adults would allow children^b^			
Adult screen time behaviour (≤2 h/day)	Odds ratio (95 % CI)	*p*-value	Odds ratio (95 % CI)	*p*-value
	School day		Weekend day	
Work day	0.45 (0.20–1.00)	0.052	0.33 (0.14–0.75)	0.008
Non-work day	0.45 (0.20–0.99)	0.046	0.67 (0.31–0.68)	0.317

^a^2013 Australian Health and Social Science panel study, Rockhampton

^b^Reference category: > 2 h/day

## Discussion

This study investigated adult views on screen time restrictions for children, and the association with adults’ own screen time behaviour. Associations were explored in the entire adult sample, and specific adult groups (men, women, parents, non-parents). Findings showed that most adults think it is appropriate to restrict children’s screen time to ≤ 2 h/day but few adults themselves adhered to ≤ 2 h/day of leisure-related screen time. Similar studies [8, 15] have shown that although parents are generally well informed about the negative impacts of high screen use and the need to restrict screen time in children, many are high screen users themselves. An Australian study [16] found that parents who did not incorporate their knowledge and awareness about healthy behaviour into their daily lives attributed their failure to challenges and barriers, such as lack of time and pressure from their children as a result of TV advertising and child peer pressure. Several studies [17–19] have shown that adult rules on screen use can effectively deter children from participating in excessive TV viewing and computer use. However, it is not just the presence of screen time rules and restrictions that is important but also the support through adult modelling of low screen use [18].

Adults who spend ≤ 2 h/day in leisure-related screen time were less likely to allow children > 2 h/day of TV viewing and/or computer use; the association did not differ between men and women, and parents and non-parents. This finding is consistent with the results from similar studies [7, 20] showing that parents who model low screen time are more likely to impose stricter screen time rules on their children. In contrast, if parents are high screen users themselves, their efforts to impose screen time restrictions for children are more likely to fail, especially when children and parents are watching TV together [10].

To our knowledge, this is the first study to investigate adult views on screen time restrictions for children and their association with adults’ own screen time behaviour. Another novel aspect in this study was the focus on the perceptions of adults beyond solely parents. Other methodological strengths of this study include the use of a large random population sample gathered across all of Australia, the use of a validated measure of adult screen time behaviour, and the distinction between weekday and weekend day screen time. This study also had limitations. Most participants were older, and although 81 % of adults were parents, fewer adults were parents of young children. Therefore, findings may not be generalisable to parents of younger children. Also, the parental status measure (parents versus non-parents) may have been sub-optimal as sometimes non-parents also undertake child caring roles, and therefore play a similar role to parents. If many non-parents in this sample were child carers, it may have influenced the results. Moreover, the screen time measures used in our study captured solely TV viewing and computer use for fun. Smartphone and tablet use was not specifically assessed; however, their use has also become an integral part of families’ life [21]. Hence, adult screen time behaviour and the proposed screen time for children may have been underestimated. Finally, we assessed adult views on screen time restrictions for children. From this we cannot infer what screen time restrictions adults actually apply in children. However, adults’ views on screen time restrictions for children presented in this study may reveal common social norms on how much screen time is appropriate for children, and these may influence children’s actual screen time. Given that adults play a major role in limiting children’s screen time, their screen time-related attitudes, knowledge and behaviour it is worth exploring further in future research. For example, future studies may explore reasons why adults think it is important to limit children’s screen time to ≤ 2 h/day although they themselves exceed to this limit. Such information may help target particular parent populations and design more effective family-based interventions to reduce screen-based activities in children. Interventions in this area may incorporate strategies to reduce screen time behaviour in both children and parents.

## Conclusions

Most adults think it is appropriate to limit children’s screen time to the recommended ≤ 2 h/day, but few adults themselves adhere to this screen time limit. Adults who engage ≤ 2 h/day in leisure-related screen time would allow children significantly less time for TV viewing and computer use. Effective interventions to reduce screen time in children may require not only the implementation of screen time rules and restrictions, but also adult modelling of less screen use.

## Abbreviations

CI: confidence interval
ICC: intra-class correlation coefficient
OR: odds ratio
SD: standard deviation
TV: television
